# Prevalence of aspiration pneumonia among stroke patients in Ethiopia: A systematic review and meta-analysis

**DOI:** 10.1371/journal.pgph.0004869

**Published:** 2025-07-17

**Authors:** Assefa Andargie Kassa, Getahun Gebre Bogale, Mekuanint Taddele, Tilahun Degu Tsega, Abebaw Molla, Wolde Melese, Segenet Zewdie

**Affiliations:** 1 Department of Public Health, College of Medicine and Health Science, Injibara University, Injibara, Ethiopia; 2 Department of Health Informatics, School of Public Health, Asrat Woldeyes Health Sciences Campus, Debre Berhan University, Debre Birhan, Ethiopia; 3 Department of Pharmacy, College of Medicine and Health Science, Injibara University, Injibara, Ethiopia; PLOS: Public Library of Science, UNITED STATES OF AMERICA

## Abstract

Aspiration pneumonia (AP) is a serious complication among stroke patients, increasing the risk of poor outcomes and death. Although a previous review in Ethiopia attempted to estimate its burden, it had methodological limitations. This study aimed to provide an updated and comprehensive estimate of the pooled prevalence of AP among stroke patients in Ethiopia. A systematic review and meta-analysis were conducted following PRISMA guidelines. Relevant studies were identified from electronic databases and grey literature. Data were analyzed using STATA version 17 with the *metaprop* package. A random-effects model with Freeman–Tukey double arcsine transformation was applied. Heterogeneity was assessed using I² and Cochran’s Q test. Subgroup analysis, meta-regression, and leave-one-out sensitivity analysis were conducted. Publication bias was evaluated using funnel plot symmetry and Egger’s test. A total of 27 studies involving 7,120 stroke patients were included. The pooled prevalence of AP was 24.26% (95% CI: 20.76%–27.95%), with significant heterogeneity (I² = 91.96%; p < 0.001). Subgroup analysis showed significant regional variation, with the highest prevalence in Harari (39.48%) and the lowest in Addis Ababa (16.31%). Meta-regression showed no significant associations with study year, sample size, sex proportion, ischemic stroke proportion, or mean age. Sensitivity analysis confirmed the robustness of the findings. No publication bias was detected. Aspiration pneumonia affects nearly one in four stroke patients in Ethiopia, with notable regional variation, highlighting the need for targeted prevention strategies.

**Registration (PROSPERO):** CRD42024498777

## Introduction

Stroke is a major global health concern, contributing significantly to morbidity and mortality rates worldwide. In 2019, it accounted for 6.2 million deaths and 139.4 million disability-adjusted life years (DALYs), making it the second most common cause of death and the third most common cause of DALYs [1]. The total value of lost welfare due to stroke worldwide in 2019 was $2059.67 billion, equivalent to 1.66% of the global gross domestic product (GDP) [2]. Based on a review study on incidence and prevalence of stroke in Africa, the unstandardized annual incidence rate of stroke ranges from 25–260 per 100,000 from 1973 to 2013 [3]. On the other hand, a systematic review and meta-analysis published in 2023 reported a crude incidence rate of 106.5 per 100,000 persons [4]. Another study reported that the in-hospital mortality from stroke was 22% in Sub-Saharan Africa [5].

In Ethiopia, the burden of stroke is on the rise, with an increasing number of individuals affected by this debilitating condition [6]. Reports on in-hospital mortality from stroke slightly vary in Ethiopia. A systematic review and meta-analysis reported 14.03% [7], and another reported 18% in-hospital stroke mortality in Ethiopia [8].

In Ethiopia, the management of acute stroke is guided by the National Guideline on Major Non-Communicable Diseases, developed by the Federal Ministry of Health in 2016 [9]. This guideline provides standardized recommendations for the diagnosis and treatment of stroke, with an emphasis on early recognition, clinical stabilization, imaging-based classification, and appropriate pharmacological interventions. However, implementation remains challenged by limited diagnostic infrastructure, delayed patient presentation, and inadequate access to thrombolytic therapy and rehabilitation services. Strengthening stroke care pathways and health system capacity is essential to improve clinical outcomes in the Ethiopian context.

Despite advancements in stroke management, stroke patients often face a multitude of complications post-stroke, which can profoundly impact their quality of life and increase healthcare burden. Aspiration pneumonia (AP), increased intracranial pressure, seizure, deep vein thrombosis, hospital acquired infections and electrolyte imbalance are among the common complications experienced by stroke patients [3,10].

AP which is the most serious type of complication, refers to the microaspiration of bacterially rich gastrointestinal or oropharyngeal secretions into the lungs in amounts high enough to cause inflammation of the alveoli and systemic circulation [11–13]. Impaired swallowing (dysphagia), gastrointestinal disorders, dentition, impaired consciousness, impaired cough reflex, and age are common risk factors associated with aspiration pneumonia [11,13–15]. Patients who have had a stroke are more likely to experience dysphagia and impaired consciousness as a result of the neurological damage caused by the event. Dysphagic stroke patients run the danger of aspirating stomach contents. AP is more prevalent among dysphagic individuals [16–19]. Moreover, stroke induced immunosuppression; driven by sympathetic nervous system activation, impaired immune cell function, elevated cortisol levels, and increased cell apoptosis; heightens the risk of developing AP after stroke [20–24].

AP poses significant burdens for stroke patients, impacting their clinical outcomes and overall quality of life. The incidence of aspiration pneumonia in stroke patients ranges from 3.9% to 12%, contributing to increased morbidity and mortality rates [24,25]. Hospitalization from both community-acquired and hospital-acquired pneumonia is frequently caused by aspiration pneumonia [26,27]. Evidences show that post stroke pneumonia is mostly associated with aspiration [28] where 3% to 50% patients with stroke may develop AP [13].

A previous systematic review on aspiration pneumonia (AP) among stroke patients in Ethiopia has been published [29]; however, it is limited by major methodological flaws. It included studies with nonspecific outcomes (e.g., pneumonia and stroke-associated pneumonia) [30,31], used data from general medical admissions instead of stroke-specific populations [32], and incorporated multiple publications from similar datasets [33,34]. Several prevalence estimates were inaccurately extracted, and at least five eligible studies were missed. Our review addresses these issues through rigorous methodology and includes six additional recent studies, offering a more accurate and comprehensive synthesis of the evidence on AP among stroke patients in Ethiopia.

This review aims to estimate the prevalence of AP among stroke patients in Ethiopia by integrating the body of available knowledge. Studying aspiration pneumonia among stroke patients is crucial for reducing its incidence, improving patient outcomes, and optimizing healthcare resources. It leads to better preventive strategies, enhanced rehabilitation techniques, and overall improved quality of life for stroke patients.

## Methods

### Protocol and registration

This systematic review and meta-analysis adhered to the Preferred Reporting Items for Systematic Reviews and Meta-Analyses (PRISMA) guidelines [35] (S1 Checklist, S2 Checklist). To ensure transparency and pre-defined methodological rigor, the study protocol was prospectively registered in the International Prospective Register of Systematic Reviews (PROSPERO) under registration number CRD42024498777.

### Eligibility criteria

Inclusion criteria were defined using the Condition, Context and Population (CoCoPop) framework [36].

- **Condition**: The primary condition is aspiration pneumonia (AP). We included all studies reporting AP as a primary outcome or a complication among individuals diagnosed with stroke. Aspiration pneumonia was defined based on clinical, radiological, or diagnostic criteria as reported by the original studies.
- **Context**: Studies conducted within the geographical boundaries of Ethiopia, regardless of healthcare setting.
- **Population**: Studies involving adult patients (aged ≥18 years) who had been clinically diagnosed with stroke, including ischemic stroke, hemorrhagic stroke, or unspecified types. Studies were eligible regardless of the sex of participants, presence of comorbidities, or stroke severity.
- **Study Types**: We included observational studies that reported the prevalence of aspiration pneumonia, including: Cross-sectional studies, Prospective or retrospective cohort studies and Case-control studies.
- **Outcome Measure**: Studies must have reported prevalence data on aspiration pneumonia among stroke patients, either as a primary outcome or as extractable from the study results.
- **Time Frame**: No restriction was applied on the year of publication. Studies published until May 10, 2025 were included.
- **Language:** We did not apply any language restriction during the literature search. However, all eligible studies identified and included in the final review were published in English.

Whereas, studies conducted among patients with a specific condition, studies focusing on pediatric populations or non-stroke-related causes of pneumonia, case reports, reviews, editorials, or commentaries that do not provide original data and studies that do not separate data on aspiration pneumonia from other types of pneumonia in stroke patients were excluded. In cases of multiple publications using the same dataset, only the most complete and recent version was included.

### Search strategy

A comprehensive search strategy was developed to identify relevant studies. Databases such as PubMed, Scopus, Cumulative Index to Nursing and Allied Health Literature (CINAHL), Semantic scholar, crossref, and Google scholar were systematically searched. We used the Harzing’s Publish or Perish (Windows GUI Edition) Version 8.2.3944.8118 software to search Scopus, Semantic Scholar, crossref and Google Scholar databases. The search strategy included Medical Subject Headings (MeSH) terms and keywords related to stroke, aspiration pneumonia, and Ethiopia. The search strings were developed using the ‘AND’ and ‘OR’ Boolean operators. Further studies were sought using a snowballing process from references of primary studies. The search was conducted from inception to May 10, 2025 for all databases, with no language or publication status restrictions applied. Filters were applied for human species and adult (18 + years). The search strategy for PubMed is presented below and others are presented in a supplementary file (S1 Text).

((stroke[mesh] OR stroke[ti] OR “post-stroke”) AND (complication OR sequelae OR comorbidity OR Outcome* OR Pneumonia OR “aspiration pneumonia”)) AND (Ethiopia) Filters: Humans, Adult: 18 + years

### Study selection

Search results from databases were exported to EndNote 20.5 (Clarivate LLC, 2022, US) and duplicates were identified. Two independent reviewers (AAK and SZ) conducted the screening process. Titles and abstracts were initially screened for eligibility, followed by a full-text assessment of potential studies. Inclusion decisions were based on predefined criteria, with disagreements resolved through consensus.

### Quality assessment

The methodological quality of included studies was assessed using the Joanna Briggs Institute (JBI) quality assessment tool for studies reporting prevalence data [36]. This tool evaluates the methodological rigor of observational studies based on criteria related to sample representativeness, study design, data collection methods, and statistical analysis in nine items. Each of the nine items in the checklist was scored as “Yes” (1 point), “Unclear” (0 points), or “No” (–1 point). A total score was calculated for each study and converted into a percentage. Based on the percentage score, studies were classified as high quality (≥ 80%), Moderate quality (60–79%) and Low quality (< 60%) (S2 Text). The quality assessment was done by two authors (TDT and WM) and discordances were resolved through discussion.

### Data extraction

Data extraction was independently performed by two reviewers (AAK and GGB) using a format on Excel spreadsheet with any discrepancies resolved through discussion. The extraction format included study characteristics (author, year of publication/posting, publication status, journal/data base, study setting, study duration, study design, data source, primary outcome and sample size), participant characteristics (mean age, proportion of male and female, proportion of urban and rural, proportion of ischemic vs hemorrhagic stroke) and study outcome (effect size) aspiration pneumonia prevalence (S2 Table). Missing data in the included studies were assessed, and where possible, study authors were contacted to obtain additional information. In cases where the mean age was not provided, we followed the recommendation of Hozo et al. and converted to the mean age [37]. In the meta-analysis, only studies with complete outcome data were included.

### Data synthesis and analysis

A narrative synthesis of included studies was conducted to summarize the findings related to post-stroke aspiration pneumonia among stroke patients in Ethiopia. The meta-analysis was performed using STATA 17 (StataCorp LLC, College Station, Texas 77845 USA). The *metaprop* package was employed to pool prevalence estimates and calculate corresponding 95% confidence intervals, accounting for between-study heterogeneity using a random-effects model. Quantitative data synthesis utilized a random-effects meta-analysis model with the Restricted Maximum Likelihood (REML) between studies variance estimation technique, considering potential heterogeneity among studies. The Freeman–Tukey transformation was employed as a variance-stabilizing transformation. This transformation was preferred for its ability to address both the problem of confidence limits outside the [0,100] range and that of variance instability [38]. To facilitate interpretation, we reported the back transformed proportions in percentage. Heterogeneity was assessed using the I^2^ statistic and test of homogeneity of effect sizes was done using the Cochran’s homogeneity test (Q statistic) at the 0.05 significance level. Subgroup analyses were conducted based on region, study design, data source and publication status. A random effects meta-regression was performed using sample size, year of publication, proportion of male patients, proportion of ischemic stroke, and mean age. Tables, figures and forest plots were used to display the results.

### Publication bias

Publication bias was assessed using funnel plots and asymmetry was tested using Egger’s regression test with a 0.05 significance level [39].

### Sensitivity analysis

Sensitivity analyses were conducted to explore the impact of methodological quality and study design on overall results. We performed the leave-one-out analysis to check the influence of individual studies on the pooled effect size.

### Ethical considerations

Since the study analyzed data that was made available to the public, ethical approval was not considered necessary.

## Results

### Study selection

**Fig 1:**
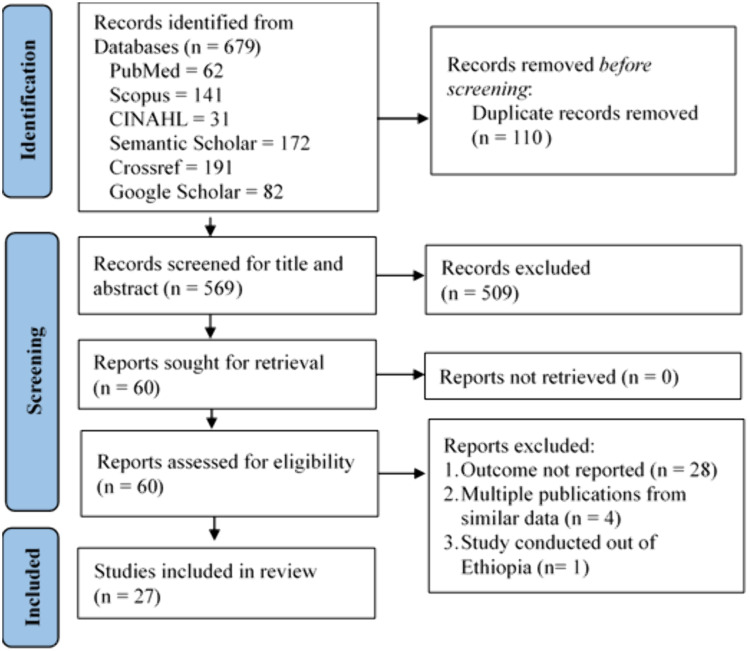
PRISMA flow diagram used to select studies for the systematic review and meta-analysis of the prevalence of aspiration pneumonia among stroke patients in Ethiopia.

A total of 679 records identified from database searches. After removing 110 duplicate records, we screened 569 records for titles and abstracts (S1 Table). We sought retrieval of full texts for 60 records and each one were assessed for eligibility. Finally, the review included 27 studies. We looked through all of the papers that cited the studies that were first included, as well as the references of the included studies. However, these tracking efforts did not turn up any more article that meet the inclusion criterion (Fig 1).

### Study characteristics

A total of 27 studies conducted between 2015 and 2025 were included in this systematic review and meta-analysis. The studies were geographically diverse, covering multiple regions of Ethiopia: Amhara (n = 9) [40–48], Oromia (n = 9) [49–57], Tigray (n = 3) [34,58,59], Harari (n = 3) [60–62], Addis Ababa (AA) (n = 2) [63,64], and Sidama (n = 1) [65]. Regarding study design, retrospective cross-sectional (RCS) [34,40,43,48,50,52,55,60,61,63] and retrospective cohort (RC) [42,44–47,53,59,62] studies were the most commonly employed designs, followed by prospective cohort (PC) [51,54,58,64], cross-sectional (CS) [41,49,57,65] and case-control [56] studies. The majority of studies utilized secondary data sources (patient records) [34,40,42–46,48–50,52–56,59–63,65], while a few incorporated primary and institutional records [41,47,51,57,58,64].

The combined sample size across all included studies was 7,120 patients. Sample sizes ranged from 71 [64] to 597 [43].

The primary outcomes assessed were diverse, including in-hospital mortality, stroke complications (e.g., aspiration pneumonia, renal dysfunction), stroke management and outcomes, and clinical characteristics such as risk factors and stroke type distribution. Only two studies reported aspiration pneumonia as their primary outcome [44,48] (Table 1).

**Table 1:** The characteristics of studies included in the systematic review and meta-analysis on the prevalence of aspiration pneumonia among stroke patients in Ethiopia.

Author (year)	Publication Status	Region	Study Design	Data source	Sample size	Primary outcome studied	Quality (%)	Rating
Abdella (2019)	Pre-Print	Amhara	RCS	PR	151	In-hospital death	77.78	Moderate
Adem (2023)	Published	Harari	RCS	PR	112	In-hospital death	55.56	Low
Asgedom (2020)	Published	Tigray	RCS	PR	216	Medical complications, death	88.89	High
Asres (2020)	Published	AA	RCS	PR	170	Stroke prevalence, nursing management and outcomes	55.56	Low
Ayehu (2022)	Published	Amhara	CS	PRI	554	Risk profile, and clinical presentation	66.67	Moderate
Ayele (2023)	Published	Harari	RCS	PR	290	Poor treatment outcome	66.67	Moderate
Bekele (2023)	Published	Oromia	CS	PR	135	Stroke treatment outcome	55.56	Low
Beyene (2021)	Published	Oromia	RCS	PR	153	Stroke treatment outcome	55.56	Low
Fekadu (2019)	Published	Oromia	PC	PRI	116	Stroke management and complications	88.89	High
Gadisa (2020)	Published	Oromia	RCS	PR	111	Stroke treatment outcome	88.89	High
Gidey (2023)	Published	Tigray	PC	PRI	272	Stroke characteristics, risk factors and mortality	66.67	Moderate
Greffie (2015)	Published	Amhara	RC	PR	98	Clinical pattern, risk factors and outcomes of stroke	66.67	Moderate
Gufue (2020)	Published	Tigray	RC	PR	503	Stroke survival	88.89	High
Kefale (2020)	Published	Amhara	RCS	PR	597	Stroke treatment outcomes	88.89	High
Lidetu (2023)	Published	Amhara	RC	PR	568	Incidence of aspiration pneumonia	100.00	High
Mamushet (2015)	Published	AA	PC	PRI	71	Medical and neurological complications	55.56	Low
Mosisa (2023)	Published	Oromia	RC	PR	480	Stroke mortality	88.89	High
Mulugeta (2020)	Published	Amhara	RC	PR	162	Magnitude, risk factors and outcomes of stroke	66.67	Moderate
Teshome (2023)	Thesis	Sidama	CS	PR	266	Treatment outcome of stroke	88.89	High
Wubshet (2023)	Published	Oromia	PC	PR	153	Stroke clinical characteristics and short term outcomes	55.56	Low
Zewudie (2020)	Published	Oromia	RCS	PR	220	Stroke treatment outcome	77.78	Moderate
Abas (2024)	Published	Harari	RC	PR	395	Mortality	88.89	High
Addisu (2025)	Published	Amhara	RC	PR	278	Length of stay	66.67	Moderate
Ayehu (2025)	Published	Amhara	RC	PRI	403	Poor sleep quality	66.67	Moderate
Nigus (2024)	Published	Amhara	RCS	PR	242	Post stroke Aspiration Pneumonia	100.00	High
Nigussie (2024)	Published	Oromia	CS	PRI	200	Renal dysfunction and in-hospital mortality	66.67	Moderate
Hussein (2024)	Preprint	Oromia	CaC	PR	204	In-hospital mortality	88.89	High

Abbreviations:

AA: Addis Ababa; CS: Cross sectional; PC: Prospective cohort; PR: Patient record; PRI: Patient record and interview; RC: Retrospective cohort; RCS: Retrospective cross sectional;

### Participant characteristics

The proportion of male participants ranged from 41.7% [34] to 72.5% [50], with a pooled mean of approximately 56.1%. Urban residency varied widely ranging from 22.9% [44] to 60.1% [54], reflecting demographic heterogeneity.

The mean age of stroke patients ranged between 52.5 years [63] and 68 years [42], with the majority of studies reporting mean ages in the early to mid-60s, suggesting a relatively younger stroke population compared to global estimates.

Ischemic stroke (IS) was generally more common than hemorrhagic stroke (HS). The proportion of IS ranged from 42.9% [65] to 100% [46], while HS ranged from 0% to 57.1%. The average IS and HS proportions across studies were approximately 61.46% and 36.4%, respectively. The prevalence of aspiration pneumonia (AP) among stroke patients was reported in all included studies, with a wide range between 7.27% [55] and 42.78% [62], indicating considerable variation across settings and patient populations (Table 2).

**Table 2:** Study participant characteristics that were part of the meta-analysis and systematic review on the prevalence of aspiration pneumonia in Ethiopian stroke patients.

Author (year)	Male %	Urban %	Mean age	IS %	HS %	AP (Count)	AP %
Abdella (2019)	49.70	35.10	65.00	60.30	39.70	45	29.80
Adem (2023)	61.60	NR	60.32	43.75	56.25	39	34.82
Asgedom (2020)	41.70	46.80	61.20	55.60	44.40	92	42.59
Asres (2020)	57.10	NR	52.49	51.20	37.60	18	10.59
Ayehu (2022)	46.75	31.60	61.00	60.29	39.71	160	28.88
Ayele (2023)	62.80	29.00	54.70	58.60	41.40	110	37.93
Bekele (2023)	63.00	37.80	57.90	64.40	25.20	17	12.59
Beyene (2021)	72.50	42.00	57.30	73.20	26.80	46	30.07
Fekadu (2019)	62.90	27.60	55.14	51.70	48.30	23	19.83
Gadisa (2020)	49.50	36.00	63.40	80.20	18.00	24	21.62
Gidey (2023)	42.60	53.70	64.30	62.90	37.10	32	11.76
Greffie (2015)	46.90	55.40	68.00	69.40	30.60	19	19.39
Gufue (2020)	50.10	56.70	64.30	56.60	43.40	183	36.38
Kefale (2020)	53.10	46.60	61.08	65.70	31.50	125	20.94
Lidetu (2023)	60.21	22.90	67.00	69.01	30.99	131	23.06
Mamushet (2015)	60.60	NR	52.76	60.50	39.40	24	33.80
Mosisa (2023)	62.29	40.63	55.43	54.58	45.42	127	26.46
Mulugeta (2020)	46.30	46.30	58.30	50.00	30.00	32	19.75
Teshome (2023)	58.60	40.60	56.14	42.90	57.10	47	17.67
Wubshet (2023)	52.90	60.10	57.00	48.40	51.60	32	20.92
Zewudie (2020)	67.30	35.10	62.33	70.90	29.10	16	7.27
Abas (2024)	62.50	51.10	61.02	64.60	35.40	169	42.78
Addisu (2025)	59.70	34.50	66.07	100.00	0.00	57	20.50
Ayehu (2025)	44.00	32.27	61.30	67.70	32.30	82	20.35
Nigus (2024)	55.80	42.10	61.15	60.30	39.70	56	23.14
Nigussie (2024)	64.50	34.00	60.50	58.00	42.00	71	35.50

Abbreviations

AP: Aspiration pneumonia; HS: Hemorrhagic stroke; IS: Ischemic stroke; NR: Not reported

### Quality assessment of included studies

Of the 27 included studies, 11 were rated as high quality [34,43,44,48,51–53,56,59,62,65], 10 as moderate quality [40–42,45–47,55,57,58,61], and 6 as low quality [49,50,54,60,63,64]. Most high-quality studies provided clear definitions of the target population, employed appropriate sampling and statistical methods. Common limitations in lower-quality studies included insufficient reporting on sample representativeness and strategies to address response bias. The overall quality of the evidence was acceptable, and no study was excluded based on quality. However, study quality was explored further in sensitivity analyses (Table 1, S2 Text).

### Prevalence of aspiration pneumonia among stroke patients

**Fig 2:**
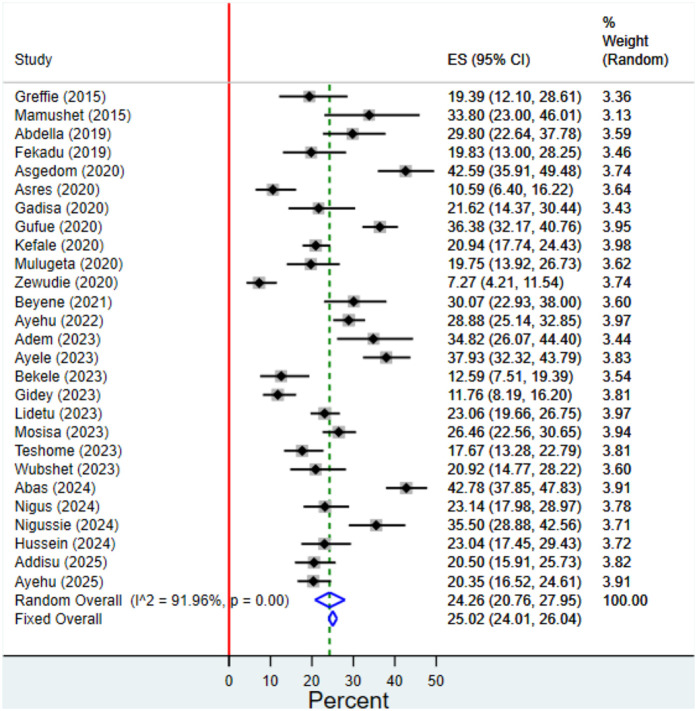
Forest plot of the pooled prevalence of aspiration pneumonia among stroke patients in Ethiopia.

The pooled prevalence of AP among stroke patients was estimated to be 24.26% (95% CI: 20.76%, 27.95%) using a random effects model. A Freeman–Tukey double arcsine transformation was applied to stabilize the variance of proportions. The analysis revealed significant between-study heterogeneity (I^2 ^= 91.96%; p < 0.001), indicating substantial variability in prevalence estimates across the included studies (Fig 2).

### Subgroup analysis

**Fig 3:**
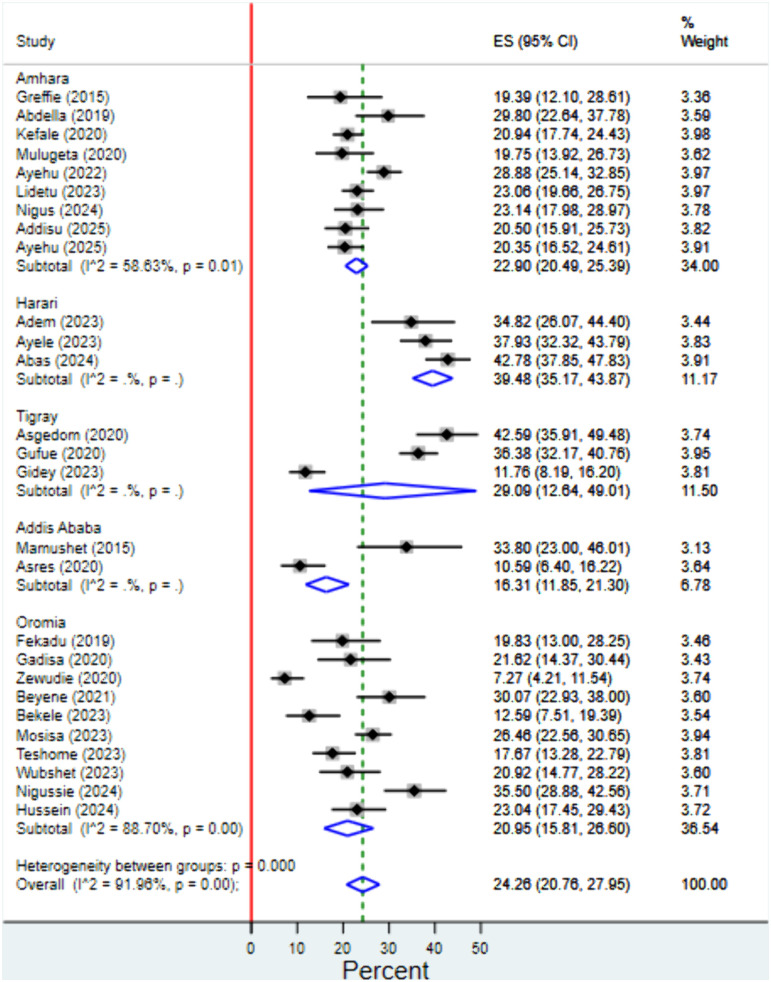
Forest plot for the sub-group analysis of the prevalence of aspiration pneumonia among stroke patients by study region in Ethiopia.

Subgroup analysis was performed to explore the source of heterogeneity by data source, study design, publication status, quality rating and region. There was no statistically significant difference in effect size across groups for data source, study design, publication status or quality rating.. Subgroup analysis by region revealed a statistically significant variation in the prevalence of AP among stroke patients. The highest pooled prevalence was observed in Harari (39.48%; 95% CI: 35.17%–43.87%), followed by Tigray (29.09%; 95% CI: 12.64%–49.01%), Amhara (22.9%; 95% CI: 20.49%–25.39%), Oromia (20.95%; 95% CI: 15.81%–26.60%), and Addis Ababa (16.31%; 95% CI: 11.85%–21.30%). The degree of heterogeneity varied across regions, with moderate heterogeneity in Amhara (I² = 58.63%) and high heterogeneity in Oromia (I² = 88.7%). Heterogeneity estimates for Harari, Tigray, and Addis Ababa could not be determined due to the limited number of studies included in each subgroup (Fig 3).

To further explore sources of heterogeneity, a meta-regression analysis was conducted using continuous study-level covariates, including study year, sample size, proportion of male participants, proportion of ischemic stroke cases, and mean age of participants. However, none of these variables showed a statistically significant association with the prevalence of aspiration pneumonia, indicating that they did not explain the observed between-study heterogeneity.

### Bias assessment

**Fig 4:**
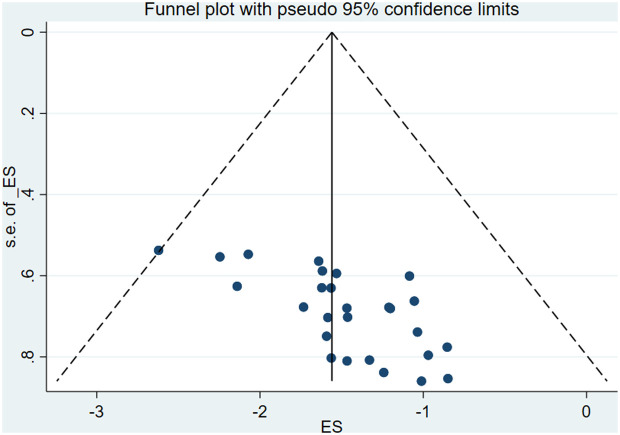
Funnel plot of included studies in the systematic review and meta-analysis on the prevalence of aspiration pneumonia among stroke patients in Ethiopia.

Assessment of publication bias using a funnel plot showed a symmetrical distribution of studies, and Egger’s regression test was not statistically significant (p = 0.454), indicating no evidence of publication bias (Fig 4).

### Sensitivity analysis

**Fig 5:**
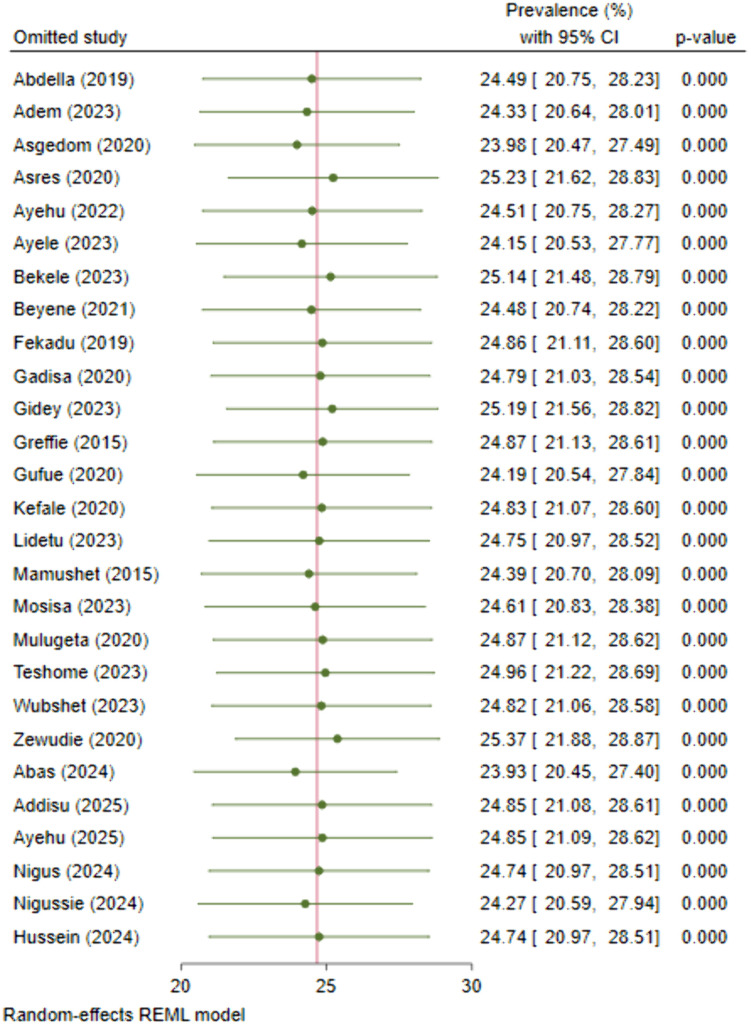
Leave-one-out sensitivity analysis of prevalence of aspiration pneumonia among stroke patients in Ethiopia.

Sensitivity analysis was performed using the leave-one-out method to assess the robustness of the pooled prevalence estimate. The results showed that no single study significantly influenced the overall pooled prevalence of aspiration pneumonia, indicating the stability and reliability of the meta-analysis findings (Fig 5).

## Discussion

This systematic review and meta-analysis aimed to determine the pooled prevalence of aspiration pneumonia among stroke patients in Ethiopia. The analysis included 27 studies encompassing a total of 7,120 stroke patients, with reported prevalence of aspiration pneumonia ranging from 7.27% to 42.78%.

In this study, the pooled prevalence estimate of aspiration pneumonia (AP) among stroke patients in Ethiopia was 24.26% (95% CI: 20.76–27.95), which is lower than the 31.65% (95% CI: 25.30–38.01) reported in a previous systematic review [29]. Notably, the confidence intervals of the two estimates do not fully overlap, suggesting a statistically meaningful difference between the findings. This discrepancy may stem from key methodological differences. The earlier review included studies with broader or unclear pneumonia definitions, such as general or stroke-associated pneumonia rather than strictly aspiration pneumonia, potentially leading to overestimation. It also contained duplicate datasets and inaccurate data extraction, and missed several eligible studies available during its search period. Our review addressed these limitations by applying stricter inclusion criteria focused solely on aspiration pneumonia, verifying data accuracy, and incorporating six additional recent studies. Consequently, our estimate likely reflects a more accurate and current burden of AP among stroke patients in Ethiopia.

This finding highlights the significant burden of AP in this population, which warrants urgent attention from healthcare providers and policymakers. The prevalence is concerning, as AP can lead to severe complications, prolonged hospital stays, increased healthcare costs, and higher mortality rates among stroke patients [66]. The finding underscores the importance of monitoring and managing this complication in stroke patients to improve outcomes and reduce mortality.

When compared to global and regional data, the prevalence of AP among stroke patients in Ethiopia is in line with a study in Japan [67]. However, it appears to be higher compared to studies conducted in other high-income countries, generally ranging from 5% to 15% [68,69]. This discrepancy may be attributed to differences in healthcare infrastructure, the availability of rehabilitation services, and early intervention strategies [70]. In many high-income settings, stroke units are well-equipped with multidisciplinary teams that provide comprehensive care, including the screening and management of dysphagia, which is a significant risk factor for AP [17].

Several factors may contribute to the high prevalence of AP among stroke patients in Ethiopia. These include limited access to specialized stroke care, inadequate screening and management of dysphagia, poor nutritional status, and suboptimal post-stroke rehabilitation services [70]. The Ethiopian healthcare system faces numerous challenges that may impact the management of stroke and its complications. These challenges include a shortage of trained healthcare professionals, limited resources, lack of standardized stroke management procedures, inadequate access to specialized care (particularly in rural areas) and insufficient infrastructure [71,72]. On the other hand, many stroke patients may not receive timely medical intervention, which increases the risk of complications including aspiration pneumonia due to delayed medical attention. High patient-to-staff ratios in Ethiopian hospitals, especially in public healthcare facilities, make it difficult for healthcare workers to provide the necessary attention and monitoring needed to prevent aspiration [73,74].

Our subgroup analysis revealed significant regional variation in the prevalence of aspiration pneumonia among stroke patients in Ethiopia. The highest prevalence was observed in Harari (39.48%), followed by Tigray (29.09%), Amhara (22.9%), Oromia (20.95%), and Addis Ababa (16.31%). This variation suggests potential differences in healthcare infrastructure, diagnostic capacity, stroke management protocols, and post-stroke care practices across regions.

The notably high prevalence in Harari may reflect either a genuinely higher burden of AP or better clinical recognition and documentation compared to other regions. For instance, Hariri region has the lowest number of hospitals compared to other regions implying that most complicated patients may be referred to these small number of hospitals [75]. Conversely, the relatively lower prevalence in Addis Ababa might be attributed to better access to acute stroke care, including early dysphagia screening and aspiration prevention strategies in tertiary hospitals.

The moderate heterogeneity observed in regions like Amhara (I² = 58.63%) and high heterogeneity in Oromia (I² = 88.7%) also indicate variability within regions, possibly due to differences in study design, sample size, or healthcare facility type (e.g., referral vs. primary hospitals). In Tigray, while the point estimate was high, the wide confidence interval suggests uncertainty, likely due to a smaller number of studies.

These findings underscore the importance of tailoring clinical and public health interventions based on local epidemiology. Regions with higher AP prevalence may benefit from strengthened capacity for early dysphagia screening, better stroke unit organization, and staff training. Future research should further explore region-specific risk factors, including clinical, demographic, and health system-related determinants, to guide more targeted and equitable stroke care strategies in Ethiopia.

### Limitations

This review has several limitations. First, most studies did not specify when aspiration pneumonia occurred, limiting interpretation of its timing relative to stroke onset. Second, there was no standardized definition of aspiration pneumonia across studies, contributing to outcome variability. Third, many studies were retrospective and reported aspiration pneumonia as a secondary outcome, which may affect data accuracy. Fourth, stroke phase and patient survival status were often unclear, making it difficult to assess population comparability. Lastly, limited data prevented subgroup analyses by stroke type, severity, or pneumonia onset, which may influence prevalence estimates.

## Conclusion

This systematic review and meta-analysis reveal a substantial burden of aspiration pneumonia among stroke patients in Ethiopia, with significant regional disparities. Addressing these disparities through targeted interventions, improved healthcare infrastructure, and increased public awareness is essential to reducing the incidence of AP and improving the overall health outcomes of stroke patients in Ethiopia. Future research should focus on identifying region-specific risk factors and developing tailored prevention and management strategies to mitigate this public health challenge.

## Supporting information

S1 Text The strategies for data bases searches. (DOCX)

S2 Text The quality assessment score of included studies. (DOCX)

S1 Checklist PRISMA 2020 Checklist. (DOCX)

S2 Checklist PRISMA 2020 for abstracts Checklist. (DOCX)

S1 Data Dataset. (XLS)

S1 Table List of studies identified in the literature search. (XLSX)

S2 Table Data extraction form. (XLSX)

## References

[1] World Health Organization. Global Health Estimates 2020: Deaths by Cause, Age, Sex, by Country and by Region, 2000-2019. 2020. https://www.who.int/data/stories/leading-causes-of-death-and-disability-2000-2019-a-visual-summary

[2] Gerstl JVE, Blitz SE, Qu QR, Yearley AG, Lassarén P, Lindberg R, et al. Global, Regional, and National Economic Consequences of Stroke. Stroke. 2023;54(9):2380–9. doi: 10.1161/STROKEAHA.123.043131 37497672 PMC7614992

[3] Akinyemi RO, Ovbiagele B, Adeniji OA, Sarfo FS, Abd-Allah F, Adoukonou T, et al. Stroke in Africa: profile, progress, prospects and priorities. Nat Rev Neurol. 2021;17(10):634–56. doi: 10.1038/s41582-021-00542-4 34526674 PMC8441961

[4] Okekunle AP, Jones S, Adeniji O, Watkins C, Hackett M, Di Tanna GL, et al. Stroke in Africa: A systematic review and meta-analysis of the incidence and case-fatality rates. Int J Stroke. 2023;18(6):634–44. doi: 10.1177/17474930221147164 36503371 PMC10313746

[5] Mohammed AS, Degu A, Woldekidan NA, Adem F, Edessa D. In-hospital mortality and its predictors among stroke patients in sub-Saharan Africa: A systemic review and meta-analysis. SAGE Open Med. 2021;9:20503121211036789. doi: 10.1177/20503121211036789 34377477 PMC8326621

[6] Feigin VL, Norrving B, Mensah GA. Global Burden of Stroke. Circ Res. 2017;120(3):439–48. doi: 10.1161/CIRCRESAHA.116.308413 28154096

[7] Tareke AA, Abate MG, Alem A, Alamneh YM, Alamneh AA, Deml YA, et al. Magnitude and Predictors of In-Hospital Stroke Mortality in Ethiopia: A Systematic Review and Meta-Analysis. Stroke Res Treat. 2022;2022:7202657. doi: 10.1155/2022/7202657 35656388 PMC9155958

[8] Alene M, Assemie MA, Yismaw L, Ketema DB. Magnitude of risk factors and in-hospital mortality of stroke in Ethiopia: a systematic review and meta-analysis. BMC Neurol. 2020;20(1):309. doi: 10.1186/s12883-020-01870-6 32814556 PMC7437163

[9] Federal Democratic Republic of Ethiopia MoH. Guidelines on clinical and programmatic management of major non communicable diseases. Addis Ababa: Ministry of Health-Ethiopia. 2016.

[10] Bhalla A, Birns J. Management of post-stroke complications. London: Springer International Publishing. 2015.

[11] Sanivarapu RR, Vaqar S, Gibson J. Aspiration Pneumonia. StatPearls. Treasure Island (FL): StatPearls Publishing. 2024. 29261921

[12] Almirall J, Boixeda R, de la Torre MC, Torres A. Aspiration pneumonia: A renewed perspective and practical approach. Respir Med. 2021;185:106485. doi: 10.1016/j.rmed.2021.106485 34087609

[13] Simpson AJ, Allen J-L, Chatwin M, Crawford H, Elverson J, Ewan V, et al. BTS clinical statement on aspiration pneumonia. Thorax. 2023;78(Suppl 1):s3–21. doi: 10.1136/thorax-2022-219699 36863772

[14] Niederman MS, Cilloniz C. Aspiration pneumonia. Rev Esp Quimioter. 2022;35 Suppl 1(Suppl 1):73–7. doi: 10.37201/req/s01.17.2022 35488832 PMC9106188

[15] Son YG, Shin J, Ryu HG. Pneumonitis and pneumonia after aspiration. J Dent Anesth Pain Med. 2017;17(1):1–12. doi: 10.17245/jdapm.2017.17.1.1 28879323 PMC5564131

[16] Marik PE. Aspiration Pneumonia and Pneumonitis. Handbook of Evidence-Based Critical Care. New York, NY: Springer New York. 2010;233–44.

[17] Chang MC, Choo YJ, Seo KC, Yang S. The Relationship Between Dysphagia and Pneumonia in Acute Stroke Patients: A Systematic Review and Meta-Analysis. Front Neurol. 2022;13:834240. doi: 10.3389/fneur.2022.834240 35370927 PMC8970315

[18] Langmore SE, Terpenning MS, Schork A, Chen Y, Murray JT, Lopatin D, et al Predictors of Aspiration Pneumonia: How Important Is Dysphagia?. Dysphagia. 1998;13:69–81. 9513300 10.1007/PL00009559

[19] Banda KJ, Chu H, Kang XL, Liu D, Pien L-C, Jen H-J, et al. Prevalence of dysphagia and risk of pneumonia and mortality in acute stroke patients: a meta-analysis. BMC Geriatr. 2022;22(1):420. doi: 10.1186/s12877-022-02960-5 35562660 PMC9103417

[20] Faura J, Bustamante A, Miró-Mur F, Montaner J. Stroke-induced immunosuppression: implications for the prevention and prediction of post-stroke infections. J Neuroinflammation. 2021;18(1):127. doi: 10.1186/s12974-021-02177-0 34092245 PMC8183083

[21] Westendorp WF, Dames C, Nederkoorn PJ, Meisel A. Immunodepression, Infections, and Functional Outcome in Ischemic Stroke. Stroke. 2022;53(5):1438–48. doi: 10.1161/STROKEAHA.122.038867 35341322

[22] Grossmann I, Rodriguez K, Soni M, Joshi PK, Patel SC, Shreya D, et al. Stroke and Pneumonia: Mechanisms, Risk Factors, Management, and Prevention. Cureus. 2021;13(11):e19912. doi: 10.7759/cureus.19912 34984111 PMC8713735

[23] Liu D-D, Chu S-F, Chen C, Yang P-F, Chen N-H, He X. Research progress in stroke-induced immunodepression syndrome (SIDS) and stroke-associated pneumonia (SAP). Neurochem Int. 2018;114:42–54. doi: 10.1016/j.neuint.2018.01.002 29317279

[24] Li D, Yuan L, Wang T, Rong Y, Li C, You M, et al. Risk factors for stroke-related pneumonia in patients with ischaemic stroke: A systematic evaluation and meta-analysis. Clin Neurol Neurosurg. 2024;246:108593. doi: 10.1016/j.clineuro.2024.108593 39426216

[25] Watila MM, Nyandaiti YW, Balarabe SA, Bakki B, Alkali NH, Ibrahim A, et al. Aspiration pneumonia in patients with stroke in Northeast Nigeria. Int J Stroke. 2013;8(4):E16. doi: 10.1111/ijs.12095 23692490

[26] Lee AS, Ryu JH. Aspiration Pneumonia and Related Syndromes. Mayo Clin Proc. 2018;93(6):752–62. doi: 10.1016/j.mayocp.2018.03.011 29730088

[27] Mandell LA, Niederman MS. Aspiration Pneumonia. N Engl J Med. 2019;380(7):651–63. doi: 10.1056/NEJMra1714562 30763196

[28] Armstrong JR, Mosher BD. Aspiration pneumonia after stroke: intervention and prevention. Neurohospitalist. 2011;1(2):85–93. doi: 10.1177/1941875210395775 23983842 PMC3726080

[29] Getie A, Ayalneh M, Bimerew M, Wondmieneh A. Aspiration pneumonia among stroke patients in Ethiopia: Systematic review and meta-analysis. Clinical Epidemiology and Global Health. 2024;28:101707. doi: 10.1016/j.cegh.2024.101707

[30] Admas M, Teshome M, Petrucka P, Telayneh AT, Alamirew NM. In-hospital mortality and its predictors among adult stroke patients admitted in Debre Markos Comprehensive Specialized Hospital, Northwest Ethiopia. SAGE Open Med. 2022;10:20503121221122465. doi: 10.1177/20503121221122465 36093420 PMC9459489

[31] Assefa M, Tadesse A, Adane A, Yimer M, Tadesse M. Factors associated with stroke associated pneumonia among adult stroke patients admitted to university of Gondar hospital, Northwest Ethiopia. Sci Rep. 2022;12(1):12724. doi: 10.1038/s41598-022-14656-2 35882874 PMC9325762

[32] Hailu A, Gidey K, Ebrahim MM, Berhane Y, Gebrehawaria T, Hailemariam T, et al. Patterns of Medical Admissions and Predictors of Mortality in Ayder Comprehensive Specialized Hospital, Northern Ethiopia: A Prospective Observational Study. Int J Gen Med. 2023;16:243–57. doi: 10.2147/IJGM.S385578 36711429 PMC9875586

[33] Weldegebreal S, Gidey K, Gidey K, Nirayo YL, Desta DM, Atey TM. Management outcome, mortality and factors associated with mortality amongst hospitalized patients with stroke: A cross sectional study. Research Square Platform LLC. 2020.

[34] Asgedom SW, Gidey K, Gidey K, Niriayo YL, Desta DM, Atey TM. Medical complications and mortality of hospitalized stroke patients. J Stroke Cerebrovasc Dis. 2020;29(8):104990. doi: 10.1016/j.jstrokecerebrovasdis.2020.104990 32689635

[35] Page MJ, McKenzie JE, Bossuyt PM, Boutron I, Hoffmann TC, Mulrow CD, et al. The PRISMA 2020 statement: an updated guideline for reporting systematic reviews. BMJ. 2021;372:n71. doi: 10.1136/bmj.n71 33782057 PMC8005924

[36] Munn Z, Moola S, Lisy K, Riitano D, Tufanaru C. Methodological guidance for systematic reviews of observational epidemiological studies reporting prevalence and cumulative incidence data. Int J Evid Based Healthc. 2015;13(3):147–53. doi: 10.1097/XEB.0000000000000054 26317388

[37] Hozo SP, Djulbegovic B, Hozo I. Estimating the mean and variance from the median, range, and the size of a sample. BMC Med Res Methodol. 2005;5:13. doi: 10.1186/1471-2288-5-13 15840177 PMC1097734

[38] Barendregt JJ, Doi SA, Lee YY, Norman RE, Vos T. Meta-analysis of prevalence. J Epidemiol Community Health. 2013;67(11):974–8. doi: 10.1136/jech-2013-203104 23963506

[39] Egger M, Davey Smith G, Schneider M, Minder C. Bias in meta-analysis detected by a simple, graphical test. BMJ. 1997;315(7109):629–34. doi: 10.1136/bmj.315.7109.629 9310563 PMC2127453

[40] Abdella SG, Gebi NB, Gerffie ES, Tamirat KS. Clinical profile, in-hospital outcome and associated factors of stroke after the start of a standard organized stroke care unit at university of Gondar hospital, northwest Ethiopia. Research Square. 2019.

[41] Ayehu GW, Yitbarek GY, Zewdie EA, Amsalu BT, Abie Y, Atlaw D, et al. Risk profile, clinical presentation, and determinants of stroke subtypes among patients with stroke admitted to public referral hospitals, Northwest Ethiopia in 2021: A cross-sectional study. Front Neurol. 2022;13:988677. doi: 10.3389/fneur.2022.988677 36388228 PMC9640987

[42] Greffie ES, Mitiku T, Getahun S. Risk factors, clinical pattern and outcome of stroke in a referral hospital, Northwest Ethiopia. Clin Med Res. 2015;4(6):182–8.

[43] Kefale B, Ewunetei A, Molla M, Tegegne GT, Degu A. Clinical pattern and predictors of stroke treatment outcome among hospitalised patients who had a stroke at Felege Hiwot comprehensive specialised hospital, northwest Ethiopia: a retrospective cross-sectional study. BMJ Open. 2020;10(12):e040238. doi: 10.1136/bmjopen-2020-040238 33384388 PMC7780509

[44] Lidetu T, Muluneh EK, Wassie GT. Incidence and Predictors of Aspiration Pneumonia Among Stroke Patients in Western Amhara Region, North-West Ethiopia: A Retrospective Follow Up Study. Int J Gen Med. 2023;16:1303–15. doi: 10.2147/IJGM.S400420 37089139 PMC10115200

[45] Mulugeta H, Yehuala A, Haile D, Mekonnen N, Dessie G, Kassa GM, et al. Magnitude, risk factors and outcomes of stroke at Debre Markos Referral Hospital, Northwest Ethiopia: a retrospective observational study. Egypt J Neurol Psychiatry Neurosurg. 2020;56(1). doi: 10.1186/s41983-020-00173-4

[46] Addisu ZD, Mega TA. Predictors of Hospital Stay After Acute Ischemic Stroke in Hospitalized Patients: Retrospective-Cohort Study. Cardiol Res Pract. 2025;2025:7598035. doi: 10.1155/crp/7598035 40364818 PMC12069843

[47] Ayehu GW, Teshome AA, Baye ND, Yitbarek GY, Amare AT, Tesfaw A. Poor sleep quality and its determinants among stroke survivors in Northwest Ethiopia. Scientific Reports. 2025;15(1):7451. 40032979 10.1038/s41598-025-92340-x PMC11876295

[48] Nigus MD, Sendek EM, Ewunetu MB, Cherkos AB, Yimer AA. Post stroke aspiration pneumonia, associated factors, and treatment outcome among stroke patients admitted to Tibebe Ghion Specialized Hospital, Bahir Dar, Ethiopia. Frontiers in Stroke. 2024;3:1410657. 10.3389/fstro.2024.1410657 PMC12802654 41542248

[49] Bekele F, Tafese L, Mohammed M, Labata BG, Chemeda GG, Fekadu G. Magnitude and predictors of stroke treatment outcomes in low resource settings: A cross-sectional study. International Journal of Africa Nursing Sciences. 2023;18:100558. doi: 10.1016/j.ijans.2023.100558

[50] Beyene N, Fanta K, Tatiparthi R. Pharmacotherapy pattern and treatment outcomes of stroke patients admitted to Jimma University Medical Center, Ethiopia. Patient Related Outcome Measures. 2021;:267–75. 34393535 10.2147/PROM.S307291 PMC8357615

[51] Fekadu G, Chelkeba L, Melaku T, Gamachu B, Gebre M, Bekele F, et al. Management protocols and encountered complications among stroke patients admitted to stroke unit of Jimma university medical center, Southwest Ethiopia: Prospective observational study. Ann Med Surg (Lond). 2019;48:135–43. doi: 10.1016/j.amsu.2019.11.003 31788240 PMC6880120

[52] Gadisa DA, Busawa GB, Gebremariam ET, Tefera GM, Belete KT, Taye GM, et al. Clinical Characteristics, Treatment Outcomes, and its Predictors Among Hospitalized Stroke Patients in Ambo University Referral Hospital, West Ethiopia: A Retrospective Hospital-Based Study. Vasc Health Risk Manag. 2021;16:591–604. doi: 10.2147/VHRM.S287465 33447039 PMC7802015

[53] Mosisa W, Gezehagn Y, Kune G, Chego M, Yigezu HF, Getnet M. Survival status and predictors of mortality among adult stroke patients admitted to Jimma University Medical Center, South West Ethiopia: A retrospective cohort study. Vascular Health and Risk Management. 2023;:527–41. 37649671 10.2147/VHRM.S399815 PMC10464890

[54] Wubshet A, Fanta K, Gemachu TD, Birhanu A, Gudina EK. Clinical characteristics and short-term outcomes of adult stroke patients admitted to Jimma Medical Center, Ethiopia: a prospective cohort study. Pan Afr Med J. 2023;44:49. doi: 10.11604/pamj.2023.44.49.37588 37070028 PMC10105338

[55] Zewudie AZ, Regasa T, Hambisa S, Nureye D, Mamo Y, Aferu T, et al. Treatment Outcome and Its Determinants among Patients Admitted to Stroke Unit of Jimma University Medical Center, Southwest Ethiopia. Stroke Res Treat. 2020;2020:8817948. doi: 10.1155/2020/8817948 33489080 PMC7790566

[56] Hussein D. Determinants of in-hospital Mortality Among Adult Stroke Patients Admitted to Public Hospital of Shashamane City West Arsi, Oromia, Ethiopia. A Two Year Unmatched Case-control Study. Research Square. 2024 15 May 2025. https://www.researchsquare.com/article/rs-5410306/v1.

[57] Nigussie EW, Abera EG, Woldemariam MB. Burden and determinants of renal dysfunction and in-hospital mortality among acute stroke patients in Ethiopia: A hospital-based observational study. Medicine (Baltimore). 2024;103(30):e39140. doi: 10.1097/MD.0000000000039140 39058854 PMC11272293

[58] Gidey K, Hailu A. A Prospective Study of Stroke Characteristics, Risk Factors, and Mortality in a Tertiary Hospital of Northern Ethiopia. Int J Gen Med. 2023;16:5051–61. doi: 10.2147/IJGM.S433353 37942476 PMC10629449

[59] Hagos Gufue Z, Gizaw NF, Ayele W, Yifru YM, Hailu NA, Welesemayat ET, et al. Survival of Stroke Patients According to Hypertension Status in Northern Ethiopia: Seven Years Retrospective Cohort Study. Vasc Health Risk Manag. 2020;16:389–401. doi: 10.2147/VHRM.S247667 33061400 PMC7533221

[60] Adem F, Mohammed B, Nigussie S. In-hospital treatment outcomes of acute stroke and determinant factors in a teaching hospital in eastern Ethiopia. SAGE Open Medicine. 2023;11:20503121221149537. 36685794 10.1177/20503121221149537 PMC9846299

[61] Ayele ZA, Molla S, Ahmed A, Worku T, Bezabih AS, Mamo BT. Poor treatment outcomes and associated factors among hospitalized patients with stroke at Hiwot Fana Comprehensive Specialized Hospital, eastern Ethiopia. Front Stroke. 2023;2. doi: 10.3389/fstro.2023.1304664 PMC12802767 41541113

[62] Abas Z, Gashaw T, Jambo A, Edessa D. In-hospital mortality and its associated factors among hospitalized stroke patients at public hospitals of Eastern Ethiopia. BMC Cardiovasc Disord. 2024;24(1):456. doi: 10.1186/s12872-024-04106-4 39192236 PMC11351025

[63] Kassaw Asres A, Cherie A, Bedada T, Gebrekidan H. Frequency, nursing managements and stroke patients’ outcomes among patients admitted to Tikur Anbessa specialized hospital, Addis Ababa, Ethiopia a retrospective, institution based cross-sectional study. International Journal of Africa Nursing Sciences. 2020;13:100228. doi: 10.1016/j.ijans.2020.100228

[64] Mamushet Y, Zenebe G, Addissie A. Medical and neurological complications among stroke patients admitted for inpatient care in addis ababa, ethiopia. Ethiop Med J. 2015;53(1):9–17. 26591287

[65] Teshome B. Treatment outcome and its determinants among adult stroke patients admitted to Hawassa University Comprehensive Specialized Hospital, Sidama Region, Hawassa Ethiopia. Hawassa University. 2023.

[66] Schwarz M, Coccetti A, Murdoch A, Cardell E. The impact of aspiration pneumonia and nasogastric feeding on clinical outcomes in stroke patients: A retrospective cohort study. J Clin Nurs. 2018;27(1–2):e235–41. doi: 10.1111/jocn.13922 28618137

[67] Watanabe S, Shimozato K, Oh‐Shige H, Umemura M, Fujiwara S, Abe Y, et al. Examination of factors associated with aspiration pneumonia following stroke. Oral Science International. 2013;11(1):15–21. doi: 10.1016/s1348-8643(13)00009-8

[68] Teh WH, Smith CJ, Barlas RS, Wood AD, Bettencourt-Silva JH, Clark AB, et al. Impact of stroke-associated pneumonia on mortality, length of hospitalization, and functional outcome. Acta Neurol Scand. 2018;138(4):293–300. doi: 10.1111/ane.12956 29749062

[69] Hassan AE, Chaudhry SA, Zacharatos H, Khatri R, Akbar U, Suri MFK, et al. Increased rate of aspiration pneumonia and poor discharge outcome among acute ischemic stroke patients following intubation for endovascular treatment. Neurocrit Care. 2012;16(2):246–50. doi: 10.1007/s12028-011-9638-0 21993605

[70] Kalkonde YV, Alladi S, Kaul S, Hachinski V. Stroke Prevention Strategies in the Developing World. Stroke. 2018;49(12):3092–7. doi: 10.1161/STROKEAHA.118.017384 30571438 PMC6942540

[71] Assefa Y, Getachew T. Successes and challenges to ensure health and wellbeing in Ethiopia. Lancet. 2022;399(10332):1283–4. doi: 10.1016/S0140-6736(22)00279-3 35294897

[72] Fekadu G. Current challenges and strategies in management and prevention of stroke. J Neurol Stroke. 2019;9:149–53.

[73] Tiruneh BT, McLelland G, Plummer V. National Healthcare System Development of Ethiopia: A Systematic Narrative Review. Hosp Top. 2020;98(2):37–44. doi: 10.1080/00185868.2020.1750323 32279644

[74] Yitbarek K, Serawit A, Medhin G, Alemayehu YK, Teklu AM, Assefa Y, et al. Capacity of the Ethiopian primary health care system to achieve universal health coverage: a primary health care progression approach. Health Policy Plan. 2023;38(4):474–85. doi: 10.1093/heapol/czad013 36760182

[75] Singh K. Health sector performance in Ethiopia: a study in regional disparities. J Afri Devt Stud. 2022;6(2):36–54. doi: 10.56302/jads.v6i2.3120

