# Supplementary material for: Prevalence of aspiration pneumonia among stroke patients in Ethiopia: A systematic review and meta-analysis
Source: PLOS Glob Public Health. 2025 Jul 17;5(7):e0004869. doi: 10.1371/journal.pgph.0004869 (PMC12270159; doi:10.1371/journal.pgph.0004869)
Supplement: S1 Text — (DOCX) [file pgph.0004869.s001.docx]

**S1 Text. Search Strategy for a systematic review and Meta-analysis of aspiration pneumonia among stroke survivors in Ethiopia.**

| **No** | **Data Source** | **Search Query** | **Search Date** | **Search Result (No of papers)** |
| --- | --- | --- | --- | --- |
|  | PubMed | ((stroke[mesh] OR stroke[ti] OR "post-stroke”) AND (complication OR sequelae OR comorbidity OR Outcome* OR Pneumonia OR “aspiration pneumonia”)) AND (Ethiopia) Filters: Humans, Adult: 18+ years | 2025-05-10 | 62 |
|  | Scopus | Title word: stroke  Keyword: (“Aspiration pneumonia” OR Complications OR outcomes) AND Ethiopia | 2025-05-10 | 141 |
|  | Semantic Scholar | stroke "aspiration pneumonia" Ethiopia | 2025-05-10 | 172 |
|  | Crossref | Title word: stroke  Keyword: (“Aspiration pneumonia” OR Complications OR outcomes) AND Ethiopia | 2025-05-10 | 191 |
|  | Google Scholar | Title word: stroke  Keyword: (“Aspiration pneumonia” OR Complications OR outcomes) AND Ethiopia | 2025-05-10 | 82 |
|  | CINAHL | “Aspiration pneumonia” AND stroke AND Ethiopia | 2025-05-10 | 31 |
