# Supplementary material for: Prevalence of aspiration pneumonia among stroke patients in Ethiopia: A systematic review and meta-analysis
Source: PLOS Glob Public Health. 2025 Jul 17;5(7):e0004869. doi: 10.1371/journal.pgph.0004869 (PMC12270159; doi:10.1371/journal.pgph.0004869)
Supplement: S2 Text — (DOCX) [file pgph.0004869.s002.docx]

S2 Text: Quality assessment score of included studies using the JBI checklist for studies reporting prevalence data.

| Study | JBI checklist items for prevalence studies | | | | | | | | | Total | % score | Rating |
| --- | --- | --- | --- | --- | --- | --- | --- | --- | --- | --- | --- | --- |
|  | Q1 | Q2 | Q3 | Q4 | Q5 | Q6 | Q7 | Q8 | Q9 |  |  |  |
| 1. Abdella (2019) | 1 | 1 | 0 | 1 | 1 | 1 | 0 | 1 | 1 | 7 | 77.78 | Moderate |
| 1. Adem (2023) | 1 | -1 | 0 | 1 | 1 | 1 | 0 | 1 | 1 | 5 | 55.56 | Low |
| 1. Asgedom (2020) | 1 | 1 | 1 | 1 | 1 | 1 | 0 | 1 | 1 | 8 | 88.89 | High |
| 1. Asres (2020) | 1 | -1 | 0 | 1 | 1 | 1 | 0 | 1 | 1 | 5 | 55.56 | Low |
| 1. Ayehu (2022) | 1 | -1 | 1 | 1 | 1 | 1 | 0 | 1 | 1 | 6 | 66.67 | Moderate |
| 1. Ayele (2023) | 1 | -1 | 1 | 1 | 1 | 1 | 0 | 1 | 1 | 6 | 66.67 | Moderate |
| 1. Bekele (2023) | 1 | 1 | 0 | -1 | 1 | 1 | 0 | 1 | 1 | 5 | 55.56 | Low |
| 1. Beyene (2021) | 1 | -1 | 0 | 1 | 1 | 1 | 0 | 1 | 1 | 5 | 55.56 | Low |
| 1. Fekadu (2019) | 1 | 1 | 1 | 1 | 1 | 1 | 0 | 1 | 1 | 8 | 88.89 | High |
| 1. Gadisa (2020) | 1 | 1 | 1 | 1 | 1 | 1 | 0 | 1 | 1 | 8 | 88.89 | High |
| 1. Gidey (2023) | 1 | 1 | 0 | 1 | 1 | 1 | 0 | 1 | 0 | 6 | 66.67 | Moderate |
| 1. Greffie (2015) | 1 | 0 | 0 | 1 | 1 | 1 | 0 | 1 | 1 | 6 | 66.67 | Moderate |
| 1. Gufue (2020) | 1 | 1 | 1 | 1 | 1 | 1 | 0 | 1 | 1 | 8 | 88.89 | High |
| 1. Kefale (2020) | 1 | 1 | 1 | 1 | 1 | 1 | 0 | 1 | 1 | 8 | 88.89 | High |
| 1. Lidetu (2023) | 1 | 1 | 1 | 1 | 1 | 1 | 1 | 1 | 1 | 9 | 100.00 | High |
| 1. Mamushet (2015) | 1 | -1 | 0 | 1 | 1 | 1 | 0 | 1 | 1 | 5 | 55.56 | Low |
| 1. Mosisa (2023) | 1 | 1 | 1 | 1 | 1 | 1 | 0 | 1 | 1 | 8 | 88.89 | High |
| 1. Mulugeta (2020) | 1 | 1 | -1 | 1 | 1 | 1 | 0 | 1 | 1 | 6 | 66.67 | Moderate |
| 1. Teshome (2023) | 1 | 1 | 1 | 1 | 1 | 1 | 0 | 1 | 1 | 8 | 88.89 | High |
| 1. Wubshet (2023) | 1 | 0 | -1 | 1 | 1 | 1 | 0 | 1 | 1 | 5 | 55.56 | Low |
| 1. Zewudie (2020) | 1 | 1 | -1 | 1 | 1 | 1 | 0 | 1 | 1 | 7 | 77.78 | Moderate |
| 1. Abas (2024) | 1 | 1 | 1 | 1 | 1 | 1 | 0 | 1 | 1 | 8 | 88.89 | High |
| 1. Addisu (2025) | 1 | 1 | 0 | 1 | 1 | 1 | 0 | 1 | 0 | 6 | 66.67 | Moderate |
| 1. Ayehu (2025) | 1 | 1 | 0 | 1 | 1 | 1 | 0 | 1 | 0 | 6 | 66.67 | Moderate |
| 1. Nigus (2024) | 1 | 1 | 1 | 1 | 1 | 1 | 1 | 1 | 1 | 9 | 100.00 | High |
| 1. Nigussie (2024) | 1 | 1 | 0 | 1 | 1 | 1 | 0 | 1 | 0 | 6 | 66.67 | Moderate |
| 1. Hussein (2024) | 1 | 1 | 1 | 1 | 1 | 1 | 0 | 1 | 1 | 8 | 88.89 | High |

Q1: Was the sample frame appropriate to address the target population?

Q2: Were study participants sampled in an appropriate way?

Q3: Was the sample size adequate?

Q4: Were the study subjects and the setting described in detail?

Q5: Was the data analysis conducted with sufficient coverage of the identified sample?

Q6: Were valid methods used for the identification of the condition?

Q7: Was the condition measured in a standard, reliable way for all participants?

Q8: Was there appropriate statistical analysis?

Q9: Was the response rate adequate, and if not, was the low response rate managed appropriately?
