# Supplementary material for: Prevalence of aspiration pneumonia among stroke patients in Ethiopia: A systematic review and meta-analysis
Source: PLOS Glob Public Health. 2025 Jul 17;5(7):e0004869. doi: 10.1371/journal.pgph.0004869 (PMC12270159; doi:10.1371/journal.pgph.0004869)
Supplement: Attachment — Submitted filename: Response to reviewers.docx [file pgph.0004869.s009.docx]

**Rebuttal letter**

**To: Editor-in-Chief**
PLOS Global Public Health
Date: September 17, 2024

Dear Editor-in-Chief,

**Subject:** **Response to Reviewer and Editorial Comments on Manuscript** **PGPH-D-24-01656**

We would like to express our sincere gratitude to you and the reviewer for the time and effort taken to review our manuscript entitled “Prevalence of aspiration pneumonia among stroke survivors in Ethiopia: A systematic review and meta-analysis” (Manuscript ID: PGPH-D-24-01656). We appreciate the valuable and constructive feedback provided, which we believe has greatly improved the quality of our paper. Below, we provide a detailed point-by-point response to the reviewers' comments. For clarity, the original comments are included in *italic*, followed by our responses in regular text, with any changes made to the manuscript clearly highlighted.

**Editorial comments:**

*As required by our policy on Data Availability, please ensure your manuscript or supplementary information includes the following:*

1. *A numbered table of all studies identified in the literature search, including those that were excluded from the analyses.  For every excluded study, the table should list the reason(s) for exclusion.  If any of the included studies are unpublished, include a link (URL) to the primary source or detailed information about how the content can be accessed.*

**Response:** Thank you. We attached an EXCEL file named “S7_Table” as a supplementary material. Within this file “Sheet 1” included all the lists of studies identified in literature review (n=460) categorized based on the data source (database). Under “Sheet 2” we included studies eligible for full text screening, their eligibility status and reason for exclusion (if a study is excluded). For unpublished works, we included the web address (URL) as a source within the bibliography.

2. *A table of all data extracted from the primary research sources for the systematic review and/or meta-analysis. The table must include the following information for each study:*

- *Name of data extractors and date of data extraction*
- *Confirmation that the study was eligible to be included in the review.*
- *All data extracted from each study for the reported systematic review and/or meta-analysis that would be needed to replicate your analyses.*

**Response:** We attached an excel file named “S8_Table” as a supplementary material that contains all elements list above.

3. *If data or supporting information were obtained from another source (e.g. correspondence with the author of the original research article), please provide the source of data and dates on which the data/information were obtained by your research group.*

**Response:** All data were extracted from the available studies. No data were required needing correspondence with authors.

4. *If applicable for your analysis, a table showing the completed risk of bias and quality/certainty assessments for each study or outcome.  Please ensure this is provided for each domain or parameter assessed. For example, if you used the Cochrane risk-of-bias tool for randomized trials, provide answers to each of the signaling questions for each study. If you used GRADE to assess certainty of evidence, provide judgements about each of the quality of evidence factor. This should be provided for each outcome.*

**Response:** We attached a word document named “S2_File” that included the quality assessment scores for each study based on the JBI critical appraisal tool.

5. *An explanation of how missing data were handled.*

**Response:** We got some missing data like percentage of urban/rural residents. However, this information has not been used in the meta-analysis, just used only in the descriptive review. Some studies missed reporting mean age (reported median age instead). In such a case we computed the mean age from the reported median age.

**Review Comments to the Author**

**Reviewer #1:**

1. *The work presented for review is interesting, addressing lesser-known non-neurological complications of stroke, such as aspiration pneumonia, which also impacts stroke treatment outcomes and mortality. The authors applied the PRISMA method, which is the appropriate approach for meta-analysis. Additionally, the choice of topic in this research is well-suited to the current needs in both medical and social contexts, particularly in African countries.
   In the first part of the article, the authors thoroughly discuss the definition of stroke and its symptoms. Although this information is standard, it may be useful in a journal aimed at epidemiologists and global health professionals. The only suggestion for this section is to include visual impairments that occur during a stroke, such as homonymous hemianopia caused by brain damage beyond the optic chiasm, which significantly increases patient disability.*

**Response:** We appreciate your deep insights to our manuscript. This will help us to improve our manuscript. We included a statement regarding visual impairment due to stroke in the revised manuscript on page 5 as follows:

“Patients with stroke may also experience visual impairments particularly those resulting from damage beyond the optic chiasm. These impairments may include homonymous hemianopia, quadrantanopia, cortical blindness, visual neglect, and diplopia. These impairments can make it difficult to read, navigate, and recognize items or people, as well as contribute to further disabilities and poor quality of life”

2. *The epidemiological data presented also requires some commentary, as the authors report very high rates. Both the incidence of stroke and mortality rates significantly exceed the parameters observed in Europe, where the incidence is approximately 90-120 cases per 100,000 inhabitants per year, and mortality rates in most European countries are lower. This disparity also warrants discussion.*

**Response:** Thank you. As we have got from literatures, this is the highest of all other incidence rates in Africa. The study was conducted in rural and urban Tanzania and reported 109 and 316 per 100,000 people in rural and urban, respectively. The figure is age standardized which is better for comparison purposes. However, we are persuaded that this data could not represent Africa as a continent. Therefore, we rewrote this part in a range than absolute number. Based on a review study on incidence and prevalence of stroke in Africa, the unstandardized annual incidence rate of stroke ranges from 25–260 per 100,000 from 1973 to 2013. On the other hand, a systematic review and meta-analysis published in 2023 reported a crude incidence rate of 106.5 per 100,000 persons (See the revised manuscript on page 4, line 74-79). This generally indicates the inconsistencies and lack of reliable data on the burden of stroke in Africa.

3. *The methodology, developed according to the PRISMA system, is well-described, and the study period between June 2008 and September 2022 is sufficiently long to be representative of the methods and assumptions presented. It is evident from the supplementary data that the authors made significant efforts to adhere to PRISMA principles, perhaps even excessively so, which, while commendable, might not have been entirely necessary.*

**Response:** Thank you.

4. *The results highlight the substantial variability in the occurrence of aspiration pneumonia, ranging from 7% to 43%, a range not typically seen in other studies. The authors rightly emphasize the significant heterogeneity of these results. Overall, the pooled proportion of 23% is notably high. One potential reason for such elevated rates, as suggested by the authors, is limited access to specialized stroke care, inadequate screening, and management of dysphagia. While this is a valid point, it also indicates a lack of standardized procedures in departments admitting stroke patients. For instance, a simple water-swallowing test conducted upon admission, which is neither time-consuming nor requires complex equipment, could effectively assess swallowing disorders and prevent the administration of oral substances, including medications and food. It is positive that the authors noticed this problem and discussed it in some detail, although it is difficult to agree with the comment that a significant problem here is the lack of expensive and requiring specialist staff techniques such as fiberoptic endoscopic evaluation. In the early stages of diagnosing stroke patients, non-instrumental methods can also be quite effective in preventing aspiration pneumonia. I would expect the authors to provide some conclusions on this matter in their discussion and conclusions.*

**Response:** Appreciated. We attempted the discussion focus on lack of infrastructure, standardized procedures, delayed medical attention and overburdened health care system. Evidences, as cited in the revised manuscript, also assure these challenges in Ethiopia. As you said, highly the lack of highly sophisticated diagnostic and treatment instruments may come after utilization of the scientifically proved simple procedures like water swallow test for the prevention of aspiration. See page 15 of the revised manuscript for modifications we made.

5. *The authors' proposed further actions to determine the causes of the large discrepancies in aspiration pneumonia rates are purposeful and interesting from a public health perspective. It would be beneficial for the authors to hypothesize possible explanations for these differences. Although there may not be precise studies on this matter, the authors, given their familiarity with their healthcare system, should be able to offer some insights. For instance, could these differences be related to the average income per person in different regions, a higher level of healthcare, variations in insurance systems, or simply the introduction of specific medical procedures in some centers but not others?*

**Response:** Thank you. We added some additional explanations for the regional variations in the prevalence of aspiration pneumonia (see page 16, line 324-329 of the revised manuscript).

6. *In summary, the authors' initiative to explore this topic should be highly regarded. The work is interesting, methodologically sound, and, after addressing the minor issues raised in this and potentially other reviews, it certainly deserves consideration for publication*

**Response:** Thank you very much indeed.

We believe that the revisions we have made address all concerns raised by the reviewers and the editorial office. We have taken care to enhance the clarity, rigor, and completeness of the manuscript. We hope that the revised version now meets the standards of **PLOS Global Public Health** and is acceptable for publication.

Thank you once again for considering our manuscript, and we look forward to your favorable response. Please feel free to contact us if further clarifications are needed.

 Sincerely,

Assefa Andargie Kassa,

Corresponding author

[assefaand@gmail.com](mailto:assefaand@gmail.com)
