# Supplementary material for: Prevalence of aspiration pneumonia among stroke patients in Ethiopia: A systematic review and meta-analysis
Source: PLOS Glob Public Health. 2025 Jul 17;5(7):e0004869. doi: 10.1371/journal.pgph.0004869 (PMC12270159; doi:10.1371/journal.pgph.0004869)
Supplement: Attachment — Submitted filename: Response_to_Reviewers_auresp_2.docx [file pgph.0004869.s010.docx]

January 10, 2025

**Editorial Office
PLOS Global Public Health**

**Subject: Rebuttal Letter for Manuscript ID PGPH-D-24-01656R1**
Title: "Prevalence of Aspiration Pneumonia among Stroke Survivors in Ethiopia: A Systematic Review and Meta-Analysis"

Dear Editor,

We sincerely appreciate the detailed and constructive feedback provided by the reviewers on our manuscript titled "Prevalence of Aspiration Pneumonia among Stroke Survivors in Ethiopia: A Systematic Review and Meta-Analysis." We have carefully considered all the comments and have revised the manuscript accordingly. Below, we provide a point-by-point response to each comment, detailing the changes made to address the concerns raised.

**Editor’s comment**

Thank you for responding to our latest query. Before we can proceed with your manuscript, please could you provide the following details:

1) In the PRISMA flowchart, you specify that originally 397 records were screened for this study. Please provide a numbered table of all those 397 studies identified in the literature search, including those that were excluded from the analyses. For every excluded study, the table should list the reason(s) for exclusion. If any of the included studies are unpublished, include a link (URL) to the primary source. An example for how we would expect a table like this to look like can be found here: <https://journals.plos.org/plosone/article?id=10.1371/journal.pone.0313866#sec017> (S2 file)

**Response:** Thank you for raising an important issue that can make our submission complete. Based on the recommended format we modified the table and attached it as S7_Table in excel format.

2) Please also update the methods section of your manuscript with an explanation of how missing data were handled.

**Response:** We included a statement how the missing data were handled in the methods section within the Data extraction sub section (line 189-194 of the track-changed manuscript).

**Reviewer #1:**

We have previously addressed all comments raised by Reviewer 1 in our initial response. We appreciate their valuable input, which significantly improved the quality of our manuscript.

**Reviewer #2:**

This is an interesting study on the incidence of aspiration pneumonia after stroke in the Ethiopian population. As I do not have the previous reviewer report, I do not know what previous comments have been made and whether they have been addressed now. So, I cannot comment on that.

Major:

1. The introduction section elaborates quite long on stroke, but should in my opinion speak more on the mechanisms of pneumonia after stroke. For example, immunosuppression after stroke is not mentioned, but is an important risk factor.

**Response:** Thank you for raising this important issue. We have reduced some unnecessary concepts related to stroke and added concepts related to aspiration pneumonia including the risk associated with immunosuppression. This can be seen on page 3 & 5 of the revised manuscript with tracked change.

2. How was aspiration pneumonia defined? What timeframe was used - first 7 days, admission, or other? What definition was used in the included studies? It is necessary for meta-analysis / pooling of data that similar definitions were used.
   Many studies exist in other populations on 'stroke-associated pneumonia', with specific definition and a time limit of 7 days after stroke. See Stroke. 2015;46:2335-2340. DOI: 10.1161/STROKEAHA.115.009617

**Response:** Thank you for this critical insight. We accept the need to define the primary outcome for a valid meta-analysis. However, most studies have not been conducted considering aspiration pneumonia as a primary outcome. They reported as additional information for other stroke related outcomes. Due to this, the majority of the studies haven’t used a diagnostic criterion for AP. We acknowledge this gap as a limitation of the study in the discussion section. We included all studies reporting AP as a complication among stroke patients admitted to hospitals. As AP can occur at any point after stroke, the time of diagnosis was not considered necessary. Studies must report the prevalence rate or provide data allowing for the calculation of prevalence.

3. Table 2: as stroke severity is a major risk factor for pneumonia, this should be incorporated in the table. Also: medical history of obstructive lung disease, smoking yes/no, dysphagia, handicap before the stroke. In line 252-257 the authors describe subgroup analysis, most important predictors for pneumonia are missing (stroke severity, dysphagia, were studies performed on a general ward or on an ICU, were patient intubated/mechanically ventilated etc).

**Response:** We thank the reviewer for raising these important points. We agree that stroke severity, dysphagia, and other predictors mentioned (e.g., medical history of obstructive lung disease, smoking status, handicap before stroke, ICU admission, and mechanical ventilation) are critical factors that may influence the risk of pneumonia following a stroke. Unfortunately, most of the studies included in our analysis did not report these variables in a consistent or sufficient manner to allow for inclusion in our tables or subgroup analyses. Specifically:

1. While stroke severity and dysphagia are recognized as major risk factors for pneumonia, these variables were not systematically reported across the studies we included. The lack of standardized reporting precluded us from including them in our tables or conducting subgroup analyses.
2. Most studies included in our review did not focus exclusively on aspiration pneumonia as a specific outcome. Instead, aspiration pneumonia was reported as one of several complications of stroke, often within the broader context of studies on stroke mortality or other outcomes. As a result, detailed information on the characteristics of stroke patients with aspiration pneumonia was not consistently available.
3. Information on whether patients were managed in an ICU or general ward, or whether they were intubated or mechanically ventilated, was rarely provided. This limited our ability to assess the influence of these factors on pneumonia risk.

We acknowledge that the absence of these critical variables is a limitation of our study and have added this point to the Limitations section of the manuscript. This gap highlights the need for future research to systematically capture and report such predictors in studies of post-stroke pneumonia.

4. Why did the authors exclude studies on a single type of stroke? were studies solely on ischaemic stroke or haemorrhagic stroke excluded? Why?

**Response:** Yes, we excluded 2 studies conducted solely among ischemic stroke patients. We did this for the following reasons:

- Stroke patients can experience diverse clinical outcomes depending on whether they suffer from ischemic or hemorrhagic strokes. Including studies limited to only one type of stroke could introduce selection bias and fail to represent the broader population of stroke survivors.
- Studies restricted to a single stroke type might introduce substantial clinical heterogeneity due to differences in pathophysiology, severity, and outcomes between ischemic and hemorrhagic strokes.
- Aspiration pneumonia risk may differ significantly between stroke types. Ischemic strokes are more common, but hemorrhagic strokes may present higher rates of aspiration pneumonia due to more severe neurological impairment.
- As the objective is to estimate the overall burden of aspiration pneumonia among all stroke survivors, studies focusing on just one subtype would not align with this goal.
- Limiting the review to studies that include both ischemic and hemorrhagic stroke patients ensures that studies are more comparable and findings are more harmonized.

5. Line 304-310: before drawing these conclusions, it should be clear whether the included population is similar in both studies regarding stroke severity, dysphagia, medical history, ICU admission etc.

   Minor
   - discussion line 286: reference 61/62 report on pneumonia in general, not specific aspiration pneumonia. see also review westendorp et al, BMC Neurology 2015, for specific rates in general wards and on ICU's

- line 121-125 a different font size is used
-line 203 'after then' --> next
- line 248-252 - description should be put in the methods section, not the results

**Response:** Thank you and we have updated the references to focus on literatures specific to aspiration pneumonia among stroke survivors. We have also addressed all editorial comments raised above (under the minor heading) which are really important to improve the quality of the manuscript.

**Additional Changes:**

In addition to addressing the reviewers’ comments, we made minor edits to improve the clarity and readability of the manuscript. These include revising typos, rephrasing sentences for clarity, removing duplicate descriptions in the results section which are already stated in the methods section (e.g., the quality assessment description in the results section), and adding essential elements in the methods section which have not been incorporated in the submitted manuscript (e.g., the number of authors participated in the quality assessment and how discordance was resolved).

In conclusion, we believe that the revisions have significantly strengthened the manuscript and addressed all the reviewers’ concerns. We have highlighted the changes in the revised manuscript for ease of review. Please let us know if further clarification or modifications are required.

Thank you for the opportunity to improve our manuscript. We deeply value the reviewers’ and editors’ efforts in providing valuable feedback. We look forward to your response.

Sincerely,

Assefa Andargie Kassa

Correspondence
