# Supplementary material for: Prevalence of aspiration pneumonia among stroke patients in Ethiopia: A systematic review and meta-analysis
Source: PLOS Glob Public Health. 2025 Jul 17;5(7):e0004869. doi: 10.1371/journal.pgph.0004869 (PMC12270159; doi:10.1371/journal.pgph.0004869)
Supplement: Attachment — Submitted filename: Response_to_Reviewers_auresp_3.docx [file pgph.0004869.s011.docx]

May 21, 2025

**Editorial Office
PLOS Global Public Health**

**Subject: Rebuttal Letter for Manuscript ID PGPH-D-24-01656R1**
Title: "Prevalence of Aspiration Pneumonia among Stroke Survivors in Ethiopia: A Systematic Review and Meta-Analysis"

Dear Editor,

We sincerely appreciate the detailed and constructive feedback provided by the reviewers on our manuscript titled "Prevalence of Aspiration Pneumonia among Stroke Survivors in Ethiopia: A Systematic Review and Meta-Analysis." We have carefully considered all the comments and have revised the manuscript accordingly. Below, we provide a point-by-point response to each comment, detailing the changes made to address the concerns raised. For a better clarity, we used blue-black font color for author response next to each reviewer comment.

**Reviewer #2:**

This is an article on an important topic that can give insight in the burden of pneumonia after stroke in Ethiopia. This would enable preventive measures and is insightful for policy makers.
The authors have addressed some of the previous comments and the article has improved by that, but there are still some major points that need to be addressed.

The line numbers refer to the line numbers in the track changes file (as no clean version is provided).

Major
- Major limitation for me is that it is not clear what population was studied. The authors describe ‘We included all studies 131 reporting AP as a complication among stroke patients admitted to hospitals’ but also name that only survivors of stroke were included. Also, no timeframe is given. For me it is unclear whether the occurrence of pneumonia during the first hospital stay for stroke was studied, or whether patients were also included with pneumonia months or even years after the stroke, for example in rehabilitation setting.

**Response:**

Thank you for this insightful and important comment. We appreciate the opportunity to clarify and improve the clarity of our manuscript.

In response to your concern, we revisited all the included studies to extract more specific information regarding the inclusion criteria of study participants and the timing of the occurrence of aspiration pneumonia. Unfortunately, we found that most of the primary studies lacked detailed information on these aspects. Specifically:

- The majority of the studies describe the population as “stroke patients admitted to hospitals” without further elaboration on the exact stage of care (e.g., acute, sub-acute, or chronic phase).
- The timeframe in which aspiration pneumonia occurred is generally not specified, and none of the studies clearly indicated whether cases of aspiration pneumonia were limited to the initial hospitalization or extended into the post-discharge or rehabilitation period.
- Additionally, most of the included studies were retrospective in design and based on medical records, which further limits the detail available regarding timing and context of pneumonia onset.
- It is also important to note that, with the exception of one study, aspiration pneumonia was not the primary outcome in these studies. Rather, it was reported as an associated complication of stroke.

Based on these findings, and to more accurately reflect the nature of the evidence synthesized, we have made the following changes in the revised manuscript:

1. We have replaced the term **“stroke survivors”** with **“stroke patients”** throughout the manuscript to better align with the terminology used in the included studies and to avoid implying that all participants had survived the acute phase of stroke.
2. We have revised the **methods** and **discussion** sections to explicitly acknowledge this limitation — namely, the lack of detailed information regarding the patient population and timing of aspiration pneumonia diagnosis in the included studies.
3. We have included this issue as a **major limitation** in the discussion, noting that this restricts our ability to make precise inferences about the timing and setting of aspiration pneumonia among stroke patients in Ethiopia.

We hope these clarifications and revisions adequately address your concern, and we thank you again for highlighting this important issue.

- Line 132 ‘was not considered necessary’. I disagree that it is not necessary. The incidence of pneumonia in stroke is the highest in the first week after stroke, when also the immune suppression is the most pronounced. Therefore it is likely that the incidence of aspiration pneumonia is different in the first week than in later course, for example weeks to months after the stroke. It is necessary to explain what population and what time phrame was used. If the duration of primary hospital admission was used, please add this to the text. If other, please explain.

**Response:**

Thank you for this important clarification. As noted in our previous response, we revisited all included studies but found that most did not specify the time frame of aspiration pneumonia occurrence. Given this, we agree that stating it “was not considered necessary” is inappropriate. We have removed this phrase and revised the manuscript to clearly acknowledge the lack of time frame data as a limitation. This change is reflected in both the methods and discussion sections.

- Line 143: what is meant by survivors of stroke? Survivor in what timeframe? How was this used for inclusion, for example, when a patient deceased in the first week because of aspiration pneumonia, was this patient included in the study or not? Could the authors explain how this was handled?

**Response**

Thank you for this important observation. As noted in our earlier response, we have replaced “stroke survivors” with “stroke patients” throughout the manuscript to avoid misinterpretation, as the primary studies did not define survival status or timeframe. Most of the included studies were retrospective chart reviews and reported aspiration pneumonia among all admitted stroke patients, including those who died during hospitalization. Therefore, patients who died due to aspiration pneumonia during the hospital stay were likely included, although this was not explicitly stated in the original studies. We have revised the manuscript accordingly and clarified this point in the discussion.

Minor
- As there is no clean version without the track changes, i cannot assess how the sentences are constructed in the clean version. In this version, there seem to be quite a lot of sentences that do not run well, for example the conclusion in the abstract, line 49 in the introduction section, line 225-227.
- Line 54 – is it ever expected?
- Line 53-54 duplicate the explanation in the lines thereafter (about the 2 types of stroke). Suggest to delete ‘a stroke … in the brain’ line 53-54.
- Line 59-68: this is a bit chaotic explanation and could be a lot shorter. For example ‘depending on the location, stroke can cause neurologic deficit such as aphasia, dysarthria, visual and motor or sensory disturbances, but also vertigo and headache.’
- Line 134-136 (‘stroke and … were included’) duplicates the sentences before, suggest to delete it.

**Response**

Thank you for highlighting these issues, and we sincerely apologize for the inconvenience caused by not providing a clean version of the revised manuscript. We have now carefully reviewed the entire document to address the issues you noted, including sentence clarity, flow, and redundancy. Specifically:

- The abstract conclusion, line 49 (introduction), and lines 225–227 have been rewritten for clarity and smoother sentence structure.
- The phrase on line 54 has been revised, and the statement in lines 53–54 regarding the two types of stroke has been deleted to avoid redundancy.
- The explanation in lines 59–68 has been shortened and reorganized for better flow, following your suggested format.
- The sentence in lines 134–136 has been removed, as it duplicates the preceding content.

A clean version of the revised manuscript is now provided to facilitate easier review. We appreciate your detailed feedback, which helped us improve the quality and readability of the manuscript.

- Line 140: suggest to update the search to look for new relevant articles.

**Response**

Thank you for the suggestion. We have updated our literature search to include studies published up to May 10, 2025, using the same search strategy across all databases. The updated search yield six (6) new studies that met our inclusion criteria. This update has been reflected in the methods and results section of the revised manuscript.

- The authors should name that no definition for AP was used, but that any definition used in the included studies was sufficient, in the limitation section.

**Response:**

Thank you for this valuable comment. We agree with your observation. In the revised manuscript, we have added a statement to the methods and **limitations section** clarifying that no uniform definition of aspiration pneumonia was applied in our review; instead, we relied on the definitions used in the individual included studies. We acknowledge this as a potential source of heterogeneity in our findings.

**Reviewer #3:**

Comment: The version I downloaded did not contain clear images, which may need to be addressed for clarity.

**Response:** We apologize for the inconvenience caused by unclear images in the previously submitted version. We have replaced all relevant figures with high-resolution versions to ensure clarity and readability. Please refer to the updated manuscript for the revised images.

Comment: For a meta-analysis consisting of 21 studies, citing more than 75 references seems excessive. A more concise reference list may improve readability.

**Response:** We appreciate the reviewer’s observation regarding the number of references. While we understand the importance of brevity for readability, we respectfully note that the current reference list includes not only the 27 primary studies included in the meta-analysis but also additional sources necessary for several reasons:

- To justify the rationale for the study by citing previous literature on stroke-associated pneumonia (SAP) and its clinical significance;
- To support the methodological approaches, including meta-analysis techniques, bias assessment tools, and subgroup/sensitivity analyses;
- To provide background on clinical guidelines, risk factors, and prevention strategies for SAP; and
- To contextualize our findings within the broader literature, including comparisons with other systematic reviews and clinical studies.

We believe that each reference adds value to the manuscript, ensuring transparency and scientific rigor. Nevertheless, we have conducted a careful review of the reference list and removed any citations deemed non-essential or duplicative. This has helped streamline the reference list while preserving the comprehensiveness and quality of the manuscript.

Comment: It would be beneficial to discuss standard care practices for stroke management and their correlation with the incidence of aspiration pneumonia. While the authors may not have access to all this data, mentioning standard care guidelines from local societies in the introduction would provide helpful context.

**Response:** We appreciate this insightful recommendation. In response, we have expanded the introduction to briefly outline standard care practices for acute stroke management, including preventive strategies for aspiration pneumonia. Where specific data were unavailable, we referred to the national Guidelines on Clinical and Programmatic Management of Major Non Communicable Diseases. This addition provides valuable context for understanding the variability in incidence across studies.

**Additionally,**

While a previous systematic review on aspiration pneumonia among stroke patients exists, we found that it has several critical methodological issues. Notably, it included studies with unrelated outcomes (e.g., general pneumonia or stroke-associated pneumonia rather than aspiration pneumonia), and reported prevalence estimates from general medical admissions rather than stroke-specific populations. Duplicate datasets were included, and several data points were inaccurately extracted. Furthermore, the review failed to include at least five eligible studies that were accessible within their declared search period. In contrast, our review addresses these limitations by applying a more rigorous and transparent methodology and includes **six additional recent studies** that provide new evidence.

Sincerely,

Assefa Andargie Kassa

Correspondence
